# The scavenging chemokine receptor ACKR2 has a significant impact on acute mortality rate and early lesion development after traumatic brain injury

**DOI:** 10.1371/journal.pone.0188305

**Published:** 2017-11-27

**Authors:** Thomas M. Woodcock, Tony Frugier, Tan Thanh Nguyen, Bridgette Deanne Semple, Nicole Bye, Matteo Massara, Benedetta Savino, Roberta Besio, Cristina Sobacchi, Massimo Locati, Maria Cristina Morganti-Kossmann

**Affiliations:** 1 National Trauma Research Institute, The Alfred Hospital, Melbourne, Australia; 2 Department of Surgery, Monash University, Melbourne, Australia; 3 Department of Pharmacology and Therapeutics School of Biomedical Sciences, The University of Melbourne, Melboune, Australia; 4 Department of Medicine, Royal Melbourne Hospital, The University of Melbourne, Melbourne, Australia; 5 Division of Pharmacy, School of Medicine, University of Tasmania, Hobart, Australia; 6 Humanitas Clinical and Research Center, Rozzano, Italy; 7 Department of Medical Biotechnologies and Translational Medicine, University of Milan, Milan, Italy; 8 Department of Molecular Medicine, University of Pavia, Pavia, Italy; 9 Istituto di Ricerca Genetica e Biomedica Milan Unit, National Research Council, Milan, Italy; 10 Department of Epidemiology and Preventive Medicine, and Australian New Zealand Intensive Care Research Centre, Monash University, Melbourne, Australia; 11 Barrow Neurological Institute, Department of Child Health, University of Arizona, Phoenix, AZ, United States of America; University of Florida, UNITED STATES

## Abstract

The atypical chemokine receptor ACKR2 promotes resolution of acute inflammation by operating as a scavenger receptor for inflammatory CC chemokines in several experimental models of inflammatory disorders, however its role in the brain remains unclear. Based on our previous reports of increased expression of inflammatory chemokines and their corresponding receptors following traumatic brain injury (TBI), we hypothesised that ACKR2 modulates neuroinflammation following brain trauma and that its deletion exacerbates cellular inflammation and chemokine production. We demonstrate increased CCL2 and ACKR2 mRNA expression in post-mortem human brain, whereby ACKR2 mRNA levels correlated with later times post-TBI. This data is consistent with the transient upregulation of ACKR2 observed in mouse brain after closed head injury (CHI). As compared to WT animals, ACKR2^-/-^ mice showed a higher mortality rate after CHI, while the neurological outcome in surviving mice was similar. At day 1 post-injury, ACKR2^-/-^ mice displayed aggravated lesion volume and no differences in CCL2 expression and macrophage recruitment relative to WT mice. Reciprocal regulation of ACKR2 and CCL2 expression was explored in cultured astrocytes, which are recognized as the major source of CCL2 and also express ACKR2. ACKR2 mRNA increased as early as 2 hours after an inflammatory challenge in WT astrocytes. As expected, CCL2 expression also dramatically increased at 4 hours in WT astrocytes but was significantly lower in ACKR2^-/-^ astrocytes, possibly indicating a co-regulation of CCL2 and ACKR2 in these cells. Conversely, *in vivo*, CCL2 mRNA/protein levels were increased similarly in ACKR2^-/-^ and WT brains at 4 and 12 hours after CHI, in line with the lack of differences in cerebral macrophage recruitment and neurological recovery. In conclusion, ACKR2 is induced after TBI and has a significant impact on mortality and lesion development acutely following CHI, while its role in chemokine expression, macrophage activation, brain pathology, and neurological recovery at later time-points is minor. Concordant to evidence in multiple sclerosis experimental models, our data corroborate a distinct role for ACKR2 in cerebral inflammatory processes compared to its reported functions in peripheral tissues.

## Introduction

Traumatic brain injury (TBI) is a leading cause of death and disability worldwide [1], yet no pharmacological intervention has been shown to be efficacious in improving patient outcomes [2]. There is therefore a need to explore alternative therapeutic targets in the brain, which could aid in the development of future pharmacological interventions. One such alternative may lie in the modulation of the brain’s inflammatory events induced by injury [3, 4].

Inflammation is a normal response to trauma and displays neuroprotective and restorative properties as it mediates the clearance of cellular debris and the release of neurotrophic factors [5]. On the other hand, it is thought that the release of immune mediators in the injured brain may contribute to secondary damage via the release of nitric oxide, reactive oxygen species, and glutamate from immune cells, such as brain-resident microglia and infiltrating leukocytes [5]. Indeed, the accumulation of activated leukocytes and microglia to the injured site after TBI is known to exacerbate tissue damage. The migration of these cells is guided via the upregulation of chemokines, a family of potent chemoattractants, which bind to multiple receptors in a redundant fashion [6]. Chemokines and their receptors are elevated acutely in TBI, leading to regulated accumulation first of neutrophils, then microglia and macrophages. The CC chemokine ligand 2 (CCL2; previously known as monocyte chemotactic protein-1, MCP-1) is of particular interest in brain trauma because it is a potent activator of the CC chemokine receptor 2 (CCR2) [7, 8], which beyond inducing a chemotactic response in macrophages, monocytes, and microglia [9, 10], may also have a direct effect on blood-brain barrier permeability [11, 12]. CCL2 is markedly increased in many models of TBI, including stab wound [13], diffuse axonal injury [14, 15], and lateral fluid percussion [16]. Additionally, in a closed head injury (CHI) model of TBI we have previously shown that CCL2 is evidently increased post-injury and precedes the accumulation of macrophages around the lesion [6]. The detrimental role of inflammatory chemokines as mediators of delayed tissue damage has been further supported by the observation that genetic deletion of CCL2, its receptor CC chemokine receptor 2, or the neutrophil CXC chemokine receptor 2, lead to attenuated leukocyte infiltration coinciding with reduced lesion volume, cell death, and sustained neurological recovery after TBI [17–20]. Combined, these studies provide compelling evidence that signalling through chemokine networks contributes to secondary tissue and neurological damage [20]. Thus, strategies aimed at controlling pro-inflammatory mediators such as CCL2 would be a potential approach to attenuate the brain’s inflammatory response and reduce the functional deficits that are commonly associated with TBI.

The atypical chemokine receptor ACKR2 (previously known as D6) differs from the classical chemokine receptor as it does not sustain directional cell migration after ligand engagement [21, 22]. Conversely, thanks to its constitutive recycling from the plasma membrane to vesicles and back to the plasma membrane [23, 24], it internalises pro-inflammatory CC chemokines, supporting their degradation and clearance from the extracellular space [25]. This scavenger function has been confirmed *in vivo*, as ACKR2^-/-^ mice, which are healthy and fertile and do not display an overtly different phenotype [26], consistently exhibit an exacerbated inflammatory response in models of skin inflammation [26–28], experimental colitis [29, 30], and infection [27, 31]. Of note, an increased concentration of inflammatory chemokines in the inflamed tissue and in draining lymph nodes was a constant finding in all experimental settings.

ACKR2 is expressed in several different tissues, most prominently on lymphatic endothelial cells in lung and skin, and on syncytiotrophoblast cells in placenta [27, 32]. Though, as mentioned, evidence of ACKR2 expression in the CNS has been reported [33, 34], its precise anatomical distribution and regulation under pathological conditions as well as its biological functions have not been defined. Considering the scavenger function of ACKR2 for inflammatory CC chemokines and their known roles in TBI, we set out to confirm the expression of ACKR2 in mouse and human brain tissue, and to determine its role after TBI using ACKR2^-/-^ mice in a model of CHI. We hypothesised that the absence of ACKR2 could result in an increased cerebral concentration of inflammatory CC chemokine CCL2 and consequently in leukocyte infiltration, potentially modifying neurological recovery after brain injury.

## Materials and methods

### Collection of human tissue samples from brain trauma victims

Human brain tissue from brain trauma victims was provided by the Australian Neurotrauma Tissue and Fluid Bank. Tissue requests were reviewed by the Neurotrauma Scientific Advisory Committee to evaluate scientific merit and to ensure that valuable CNS tissue is only provided to feasible research proposals. The use of human brain tissues for this study was approved by the Alfred Ethics Committee (Project 211/06). These 27 individuals were aged between 12 and 78 years (mean 48), and were classified by survival time as either “acute” (death occurred within 17 minutes of injury), “early” (death occurred between 30 minutes and 3 h from time of injury), or “late” (death occurred between 6 and 122 h from time of injury). The brain region was taken in proximity of the injured tissue and was identified macroscopically by a Neuropathologist. The second brain region for the late group (Late CL) was located in the contralateral brain hemisphere with no macroscopic damage detectable. Control brain samples of 10 individuals, without brain injury or other neuropathologies were obtained from the National Neural Tissue Resource Centre of Australia and denoted as “control” [35]. Procedures on human brain tissues were conducted in accordance with the Australian National Health & Medical Research Council’s National Statement on Ethical Conduct in Human Research (2007), the Victorian Human Tissue Act 1982, the National Code of Ethical Autopsy Practice, and the Victorian Government Policies and Practices in Relation to Post-Mortem Examinations. Brain tissue was collected in collaboration with the Victorian Institute of Forensic Medicine and approval obtained from the Human Ethics Committee (EC 2/2010) following formal consent from the next of kin.

### The CHI experimental model

Male C57Bl/6 mice aged 12–14 weeks and weighing ~30 g were obtained from the Alfred Medical Research and Education Precinct (AMREP) Animal Services (Melbourne, Australia) and housed on a 12 h light/dark cycle with food and water supplied *ad libitum*. ACKR2^-/-^ mice (C57Bl/6 background; first established by [26] were imported from Charles River Laboratories (Milan, Italy), and a colony was established at the Alfred Medical Research and Education Precinct animal facility. CCL2^-/-^ mice (C57Bl/6 background) were imported from The Jackson Laboratories (Bar Harbor, ME, USA), and a breeding colony was established at Alfred Medical Research and Education Precinct. For brain injury studies, a weight-drop model of CHI developed by Shohami’s group, recently described in detail by Flierl *et al* [36, 37] and published earlier by our group [6, 38], was utilised. Briefly, mice were anaesthetised by being placed in a bell jar containing diethyl ether-soaked cotton balls (AJAX). The skull was then exposed by making a longitudinal incision down the midline of the scalp. The mouse was then placed under the weight-drop machine and lined up so that the silicon impactor tip was at approximately bregma -2.00 mm, and 3 mm lateral to the midline on the left side of the head. Injury was induced by dropping the 333 g impactor from a height of 2 cm onto the exposed skull; this resulted in skull fracture and underlying cortical contusion. For analysis, only mice showing a skull fracture with underlying contusion were included in the study. The scalp incision was then sutured and the mouse returned to its cage on a heated pad to aid in recovery. Sham operated mice underwent anaesthesia and scalp incision, but were not placed under the impactor. After brain injury, mice were monitored for 1 h when first neurological testing occurred. During this time period mice were euthanized if the conditions for euthanasia were met, otherwise they were killed immediately when checked on following days. The specific criteria used to determine when animals should be euthanized were represented by prolonged apnoea immediately after brain injury despite resuscitation; absence of eating, drinking, apathy, lack of spontaneous movement, lack of body and hair care, trembling, permanently bent posture; weight loss greater than 15% at any stage after brain injury. ACKR2^-/-^ (n = 79) and WT mice (n = 72) were used for brain injury model. All mice that survived first hour survived later time points. The expected mortality rate immediately after the traumatic impact in this model is 25%. 36 ACKR2^-/-^ and 18 WT mice died immediately after brain injury without the need of euthanasia. No additional animals died in the recovery period. All mice that survived first hour survived later time points and were monitored daily for food and water intake by weighing the mice starting from the day before trauma up to final time point at 7 days to evaluate possible ongoing stress following brain injury. Mice were anaesthetized with ether in a closed container on a wire mesh over ether-soaked cotton. Anesthesia was maintained briefly with a few drops of ether on cotton in a 5 ml syringe used as a nose cone, while the skull was exposed by a longitudinal incision of the skin to allow for trauma induction. With approval by the ethics committee and facility veterinarian, no analgesics were used. Opioid may interfere with motor function and thus alter the results of the neurological tests performed at 1 h post-injury. Male mice were housed individually to prevent aggressive behaviour using cage separators. All staff members were nominated and approved by the ethical board and received training in the laboratory and by the animal facility prior to the experiments. For astrocyte cell cultures, pups (0–2 day old; 120 pups/strain) were killed by decapitation to harvest the brain and isolate cells. For mRNA and protein expression measurement, mice were killed at 2, 4, 12, and 24 h post-injury by decapitation under deep anaesthesia. For immunohistochemistry and neurological testing, mice were killed at 1, 3, and 7 days post-brain injury. The brain was rapidly removed from the skull and dissected to yield a 5 mm diameter piece of pericontusional cortex. These samples were immediately frozen and stored at -80°C until RNA or protein purification procedures were performed. Procedures on animals were carried out in accordance with the Code of Practice for the Care and Use of Animals for Scientific Purpose as stated by the National Health and Medical Research Council of Australia, and received prior approval from the Medical Research and Education Precinct Animal Ethics Committee (CHI experimental procedure: approval # E/0731/2008/M; cell culture experiments using neonate mice: approval # E/0583/2007/M).

### Neurological severity score (NSS)

Neurological impairment following experimental brain injury was assessed using a battery of 10 tests shown to discriminate sensorimotor function between WT and chemokine/chemokine receptor knockout mice [6, 20, 39] (Table 1). The trials assess aspects of injury including motor function, physiological behaviour, and alertness, each with a possible score of 0 (pass) or 1 (fail). A neurological severity score out of 10 is produced by summing all the individual test scores together, thus failure of all tasks would give a maximum score of 10, indicating severe neurological impairment. To monitor neurological recovery from injury, NSS testing was performed at 1 h after injury, then once each day for 7 days beginning 24 h after injury.

**Table 1:** Neurological severity score (NSS).

Task	NSS
Presence of mono- or hemiparesis	1
Inability to walk on a 3-cm-wide beam	1
Inability to walk on a 2-cm-wide beam	1
Inability to walk on a 1-cm-wide beam	1
Inability to balance on a 1-cm-wide beam	1
Inability to balance on a round stick (0.5 cm wide)	1
Failure to exit a 30-cm-diameter circle (for 2 min)	1
Inability to walk straight	1
Loss of startle behavior	1
Loss of seeking behavior	1

### Tissue collection and processing

For histological staining and immunohistochemical experiments, cohorts of mice were killed at 1, 3, or 7 days post-CHI, corresponding to the onset and peak of chemokine upregulation and macrophage infiltration. Briefly, following the induction of deep anaesthesia with intraperitoneal injection of 25% (w/v) urethane, the heart was exposed surgically and a catheter was inserted into the right ventricle. The animal was then perfused with heparinised saline (10 U/ml heparin in 0.9% saline, 120 ml/h) followed by 4% paraformaldehyde. The carotid veins were cut with micro scissors to confirm adequate perfusion of the animal. Once perfusion was complete, the brain was removed from the skull, incubated for 24 h in Bouins fixative, and stored in 70% ethanol prior to embedding and sectioning. Brains were processed in the Shandon Citadel 1000 Tissue Processor (Thermo Scientific), which dehydrated the brains through increasing concentrations of ethanol and a final immersion in xylenes over a 24 h period. Following dehydration, brains were embedded in paraffin wax and sliced into 5 μm coronal sections using a RM2235 microtome (Leica). Serial sections were mounted onto SuperFrost slides (Mikro-Glass) and were collected from approximately bregma -4.00 mm to 1.20 mm.

### Lesion volume analysis following CHI

For determination of lesion volume, sections were stained with haematoxylin and eosin (H&E) to visualise the contused cortical tissue and distinguish from healthy tissue to then measure the area of the lesion in each section [6, 20]. Briefly, perfusion fixed brain sections (5 μm thick) mounted on slides were hydrated in a series of descending concentrations of ethanol and stained with haematoxylin for 5 minutes, followed by a dip in 95% ethanol containing 1% HCl, and incubation in 2% sodium bicarbonate until optimal nuclear staining was achieved. The slides were then immersed in eosin solution for 3 minutes, rinsed in water, dehydrated and coverslipped with DPX mounting media (Sigma-Aldrich). A series of 10 images for each brain were captured at 4x magnification at a sampling frequency of 40 (one every 40 sections was analyzed, approximately 200 μm apart), to encompass the entirety of the lesion. The lesion area (A) was measured on each section, and lesion volume was estimated as a product of the sum of lesion areas (∑A), the section thickness (T), and the sampling frequency (I_SF_). Lesion volume was assessed at 1, 3, and 7 days post injury in both WT and ACKR2^-/-^ mice.

### Bone histology and X-ray analysis

Bone histology and X-ray analysis were conducted on un-injured ACKR2^-/-^ and WT male C57Bl/6 mice aged 12–14 weeks and weighing ~30 g (n = 4–6 per genotype) maintained in the specific pathogen free facility at the Humanitas Clinical and Research Center, following procedures approved by the Institutional Animal Care and Use Committee in accordance with international laws. For histology analysis, after soft tissues removal, bones were fixed in 4% paraformaldehyde and processed as previously reported [40]. Briefly, fixed bones were washed in HBSS for 10 minutes, decalcified in Ion Exchange Decal Unit (Biocare Medical) for 4 h and paraffin embedded using a ASP300S fully enclosed automatic tissue processor (Leica). Three micron thick sections were obtained from the skull coronal sections using a Microm HM310 microtome (GMI). Sections were hydrated in descending concentrations of ethanol and stained with haematoxylin for 15 minutes, washed under running tap water for 10 minutes, then immersed in eosin solution for 8 minutes, rinsed in water, dehydrated and cover-slipped with Eukitt mounting media (Sigma-Aldrich). Images were captured at different magnifications. For X-ray analysis, WT and ACKR2^-/-^ mice were analysed after euthanasia using the Mx-20 multifocus digital radiography system (Faxitron Bioptics) set at 25kV for 19 seconds. The radiographic cassettes were acquired using the Kodak DirectView elite CR System K-Pacs software (Kodak). For the morphometric analysis, dorsal X-ray projections were considered. To perform the measurements a best-fit mid-sagittal line (condylobasal length, CBL) was manually drawn from the nose to the cranial base. Two transverse lines were then traced at the level of the skull inferior region near the paraoccipital process (braincase width, BW) and of the mid-vertical region at the zygomatic root of temporal bone (cranium zygomatic width, ZW), respectively. ZW, BW, CBL, area, perimeter, major axis, minor axis and circularity (1 = perfect circle to 0 = elongated shape) were evaluated using Image J. Cranium aspect ratio (ratio major axis/minor axis), roundness (1/aspect ratio) and the ZW/CBL were calculated.

### Murine primary astrocytes cell cultures

Mouse primary astrocytes were isolated from 10 neonate (0–2 days old) WT, ACKR2^-/-^, or CCL2^-/-^ mice, as previously described [39]. Briefly, the mice were decapitated, the brains sliced into left and right hemispheres, and the meninges were removed under a dissecting microscope. Brain tissue was minced and dissociated in Hank’s balanced salt solution (HBSS) containing 0.5% (w/v) trypsin for 15 minutes at room temperature. The digest was terminated by the addition of Dulbecco’s modified Eagle’s medium (DMEM) containing 0.25% (w/v) gentamycin and 10% (v/v) deactivated foetal bovine serum. The tissue sample was then mechanically dissociated in HBSS containing 1 mg/ml DNase I. Cells were resuspended in DMEM and divided into three 75 cm^2^ flasks coated with poly-l-lysine and maintained for 7–10 days to establish confluence. Contaminating microglia and oligodendrocytes were removed by vigorous mechanical agitation in HBSS to yield an astrocyte purity of > 95% as verified by staining for glial fibrillary acidic protein. Astrocytes were then trypsinized in HBSS containing 0.25% trypsin and 1 mM EDTA and counted using a haemocytometer. Finally, cells were plated out into four 75 cm^2^ flasks and cultured until confluent. qPCR and ELISA were carried out using 3 individual cell cultures per group, and each individual culture was assayed in triplicate. Prior to stimulation, confluent cultures were washed twice with HBSS and incubated for 16 h in low-serum conditions (DMEM + 1% FBS). The cells were then stimulated by the addition of DMEM containing 1% FBS and 10 ng/ml LPS (from *Escherichia coli* 0111:B4 γ-irradiated; 0.1–100 μg/ml) and incubated for 2, 4, or 12 h. At these pre-determined time points, the cells were washed with HBSS three times and then scraped off the flasks and centrifuged to yield cell pellets that were used for RNA extraction.

### RNA extraction and quantitative PCR (qPCR) analysis

Total RNA was extracted from pericontusional mouse brain cortex, human brain, and astrocyte cell cultures using the PureLink™ RNA Mini Kit (Invitrogen) and supplied protocols as previously described [35]. The samples were homogenised for 2 minutes in lysis buffer containing 1% mercaptoethanol and centrifuged at 1,360 g for 5 minutes. The samples were then diluted 1:1 with 70% ethanol and vortexed thoroughly to disperse precipitates, before being loaded into spin cartridges attached to collection tubes. These were spun at 12,000 g for 15 seconds and flow through was discarded. DNA contamination was removed by adding Purelink™ DNase (3 U/μl resuspended DNase in DNase I reaction buffer diluted in RNase free water) and incubated at room temperature for 15 minutes, followed by washing with Wash Buffer I and II. Finally, the cartridges were dried by spinning at 12,000 g for 2 minutes, and RNA was eluted by the addition of RNase-free water. Samples were stored at -80˚C until further analysis. RNA concentration and quality was measured using a NanoDrop 1000 spectrophotometer (Thermo Fisher Scientific). A 260/280 absorbance ratio of 1.8–2.0 was considered to be pure RNA. cDNA was synthesised from 1 μg of purified RNA using SuperScript III reverse transcriptase (Invitrogen) as performed earlier [34]. Briefly, the RNA was added to RNase-free water and incubated at 65°C with 50 mM oligo d(T)_20_ and 10 mM dNTPs for 5 minutes. Samples were then cooled on ice and a solution of RT buffer, 25 mM MgCl_2_, 100 mM dithiothreitol, RNase-OUT, and SuperScript III was added before incubating in the thermocycler (50°C for 50 minutes, 85°C for 5 minutes, hold at 4°C). The newly generated cDNA was then stored at -20°C in aliquots. Samples of cDNA were prepared for qPCR by adding TaqMan Universal master mix (Applied Biosystems) and the TaqMan gene expression assay for either mouse ACKR2 (CAT#Mn00445551_m1; Applied Biosystems), mouse CCL2 (CAT#Mn00441242_m1; Applied Biosystems), human ACKR2 (CAT#Hs00174299_ml; Applied Biosystems), or human CCL2 (CAT#Hs00234140_ml; Applied Biosystems) similar to a protocol used previously on these brains [34]. The samples were then loaded onto a 384-well plate in triplicate, and PCR was carried out using a 7900 Fast qPCR system (Applied Biosystems) set for 40 cycles. Analysis of the qPCR data was done using the comparative cycle threshold (Ct) method, also known as the 2^-ΔCt^ method. For mouse experiments, the calculated Ct values of samples and controls are all normalised to the Ct values of a single endogenous housekeeping gene (GAPDH, CAT#Mn99999915_g1; Applied Biosystems), and the Ct values of the samples are then compared to those of the controls. The data is shown as a fold change in expression compared to WT controls after normalisation. GAPDH was selected as the control gene because its expression did not change significantly following CHI in our model (see data at http://dx.doi.org/10.17632/fwxsg7g6rt.1). For human qPCR experiments, the Ct values were normalised to the geometric mean of Ct values from four housekeeping genes (PPIA, HMBS, UBC, GAPDH) selected on the basis of previous publications [35].

### Measurement of CCL2 by enzyme-linked immunoassay (ELISA)

Pericontusional cortex tissue was homogenised using a T10 Basic Ultra-Turrax rotor-stator (Ika) in an extraction buffer containing 100 mM Tris buffer (pH 7.4), 1% v/v Triton X-100, 10% v/v complete tablet (Roche) as described previously in this model [20]. Samples were then incubated at 4°C for 90 minutes with gentle agitation before being centrifuged at 12,000 g for 15 minutes to remove cellular debris and allow for supernatant collection. Extracted protein concentration was then measured by Bradford assay. ELISA was performed using the mouse CCL2 Quantikine kit (R&D Systems) as described previously [20]. Samples were run in duplicate, averaged, and CCL2 concentration calculated from a standard curve generated from known CCL2 concentrations, then corrected for total protein concentration.

### Statistics

Statistical analyses were carried out in SPSS v.19.0 (IBM Corporation) and graphs were produced using GraphPad Prism 5 (GraphPad Software). 1-way ANOVA was used to analyse data where there was only one independent variable, and 2-way ANOVA was used in instances where there were two independent variables (e. g. genotype and time-point). In instances where the variances of individual groups significantly differed, the data were transformed by taking a natural logarithm of the value to normalise data prior to statistical analysis. NSS data was analysed by 2-way ANOVA with time as a repeated measure. Post-hoc analysis was carried out using Tukey’s honestly significant difference (HSD) test, and in all cases statistical significance was accepted at a 95% confidence level (α = 0.05).

## Results

### ACKR2 is induced in human brain following TBI

**Fig 1:**
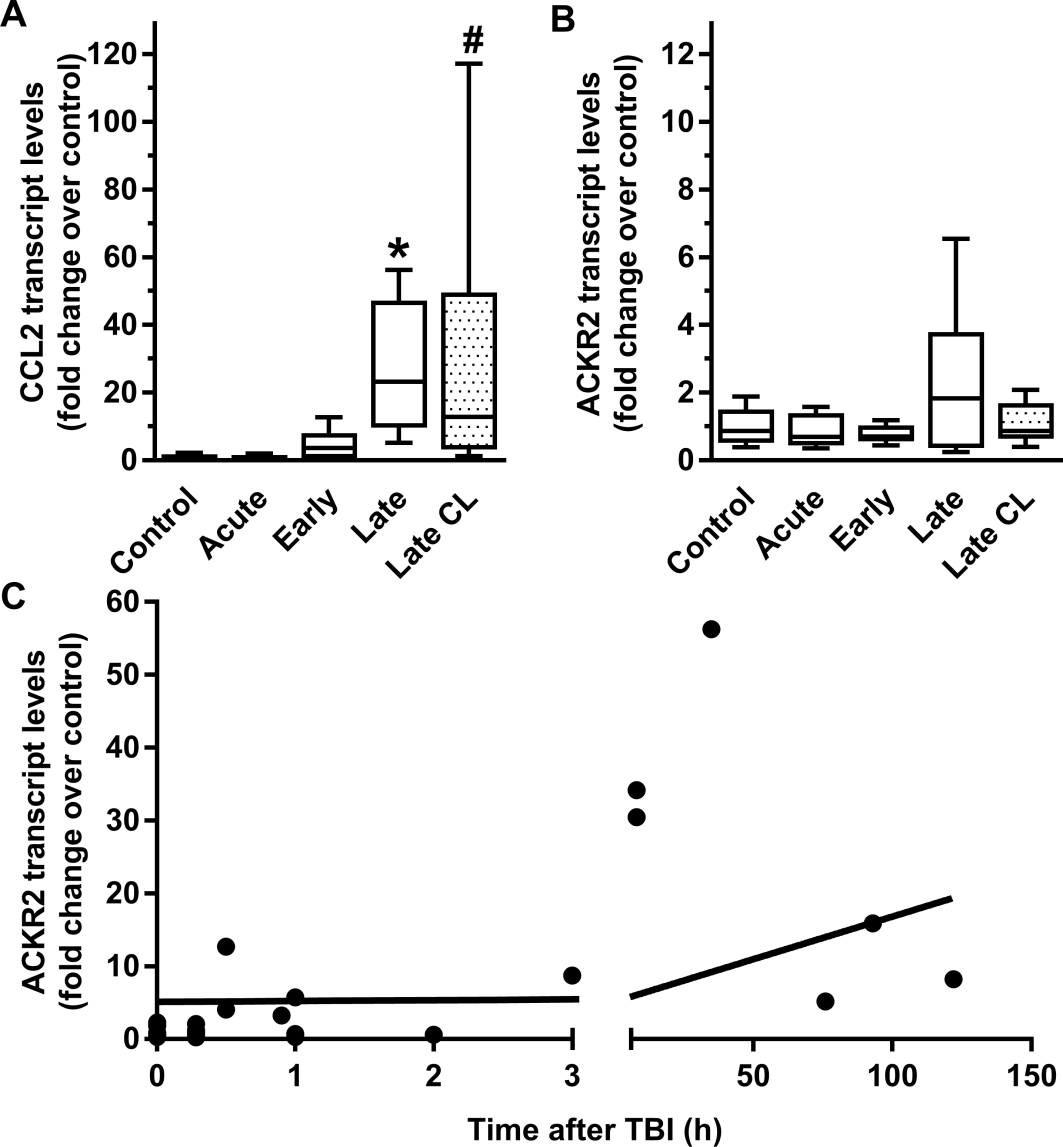
CCL2 and ACKR2 transcripts are upregulated following human brain injury. (A-B) Levels of CCL2 (A) and ACKR2 (B) transcripts were assessed by qPCR in brain tissue from TBI patients that had died < 17 minutes (acute; n = 9), < 3 h (early; n = 8), or > 6 h (late; n = 6) after injury, and in patients whose cause of death was not brain injury (control; n = 8). ANOVA reported a significant increase in CCL2 expression in the late group versus the acute group (* = p < 0.05) and in the late contralateral (late CL; dotted boxes) group versus the control group (# = p < 0.05), and no significant effect of time on ACKR2 transcript expression. (C) Correlation of ACKR2 transcript levels with time.

After TBI a significant upregulation of CCL2 mRNA was detected, with a 27-fold increase in the ‘late’ group (p < 0.01). Tukey’s multiple comparisons tests confirmed that this increase was also statistically significant over mRNA levels measured in the ‘early’ group (p < 0.05). Interestingly, we also detected a 29-fold increase in CCL2 mRNA in brain samples taken from the hemisphere contralateral to injury in the ‘late’ group of TBI victims. This increase was determined to be statistically significant versus control samples by Tukey’s post-hoc test (p < 0.05) (Fig 1A). These results are consistent with our previous observation of increased CCL2 protein concentration in human cerebrospinal fluid following TBI [6].

To investigate whether ACKR2 expression was also affected by TBI, we measured changes in ACKR2 mRNA levels in *post mortem* brain tissues in the same groups of TBI victims as well as uninjured controls (Fig 1B). Although we did not detect significant difference between the groups (p = 0.17), a clear trend towards increased levels was evident in the ‘late’ group. As survival time in this group varied from 8 to 122 h, we calculated a Pearson’s product-moment correlation coefficient to assess the relationship between ACKR2 expression and survival time. A gradual increase of ACKR2 transcript over time post-TBI was observed, with a significant positive correlation between ACKR2 expression and survival time (r = 0.61, n = 31, p < 0.001) (Fig 1C).

### ACKR2 deletion increases mortality rate and lesion volume in experimental CHI

The role of ACKR2 in TBI was further investigated by comparing a number of outcome measures in ACKR2^-/-^ (n = 79) and WT mice (n = 72) subjected to CHI. We observed a significant difference in mortality rate being 25% in WT mice, well in line with the reported 26% mortality rate for this model [36], and a striking 46% mortality in ACKR2^-/-^ mice (α-level: 0.05; p< 0.01; 2-proportion Z-test). Of note, death always occurred within a few minutes from injury.

**Fig 2:**
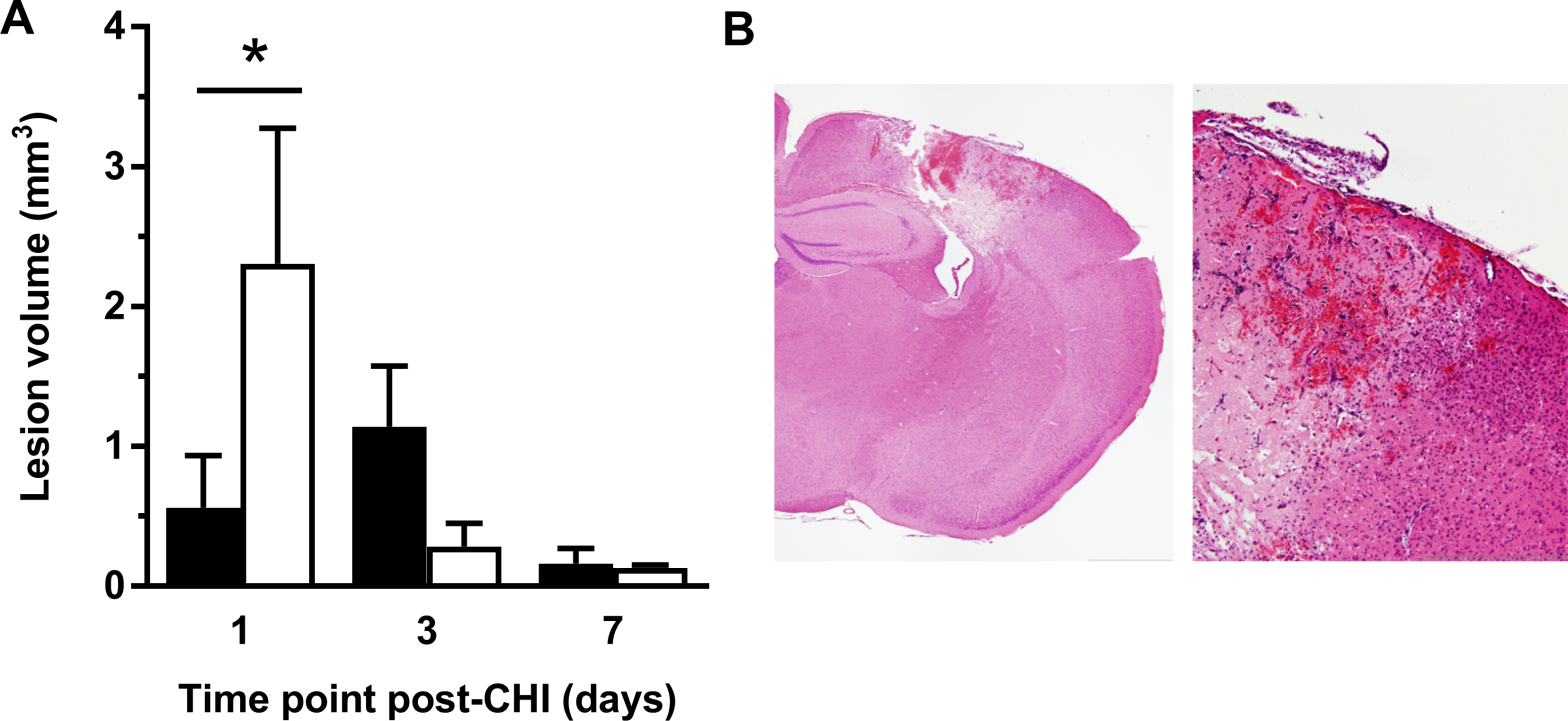
Transient increase in brain lesion volumes in ACKR2^-/-^ after CHI. (A) Brain lesion volume was assessed by H&E staining at day 1 (n = 8), 3 (n = 9), and 7 (n = 5) after CHI. ACKR2^-/-^ mice (white columns) were found to have significantly larger lesions as compared to WT mice (black columns) at day 1 post-CHI (* = p < 0.05). No significant differences were observed at later time points. ANOVA did not detect any significant effect of genotype. (B) Representative low and high magnification images of an ACKR2^-/-^ mouse brain at 24 h post-CHI.

Animals surviving brain injury were sacrificed at 1, 3, and 7 days post-CHI to analyse differences in lesion volumes on H&E stained sections (Fig 2). While there was no overall effect of genotype (p = 0.47) or interaction of time and genotype (p = 0.06), *post hoc* analysis revealed a transient, significant difference on day 1, with ACKR2^-/-^ mice having significantly larger lesions than WT (p < 0.05). Two-way ANOVA also demonstrated a significant effect of time post-CHI (p = 0.046), which represents the evolution of the lesion over 7 days.

### ACKR2 deletion has no impact on bone structure

**Fig 3:**
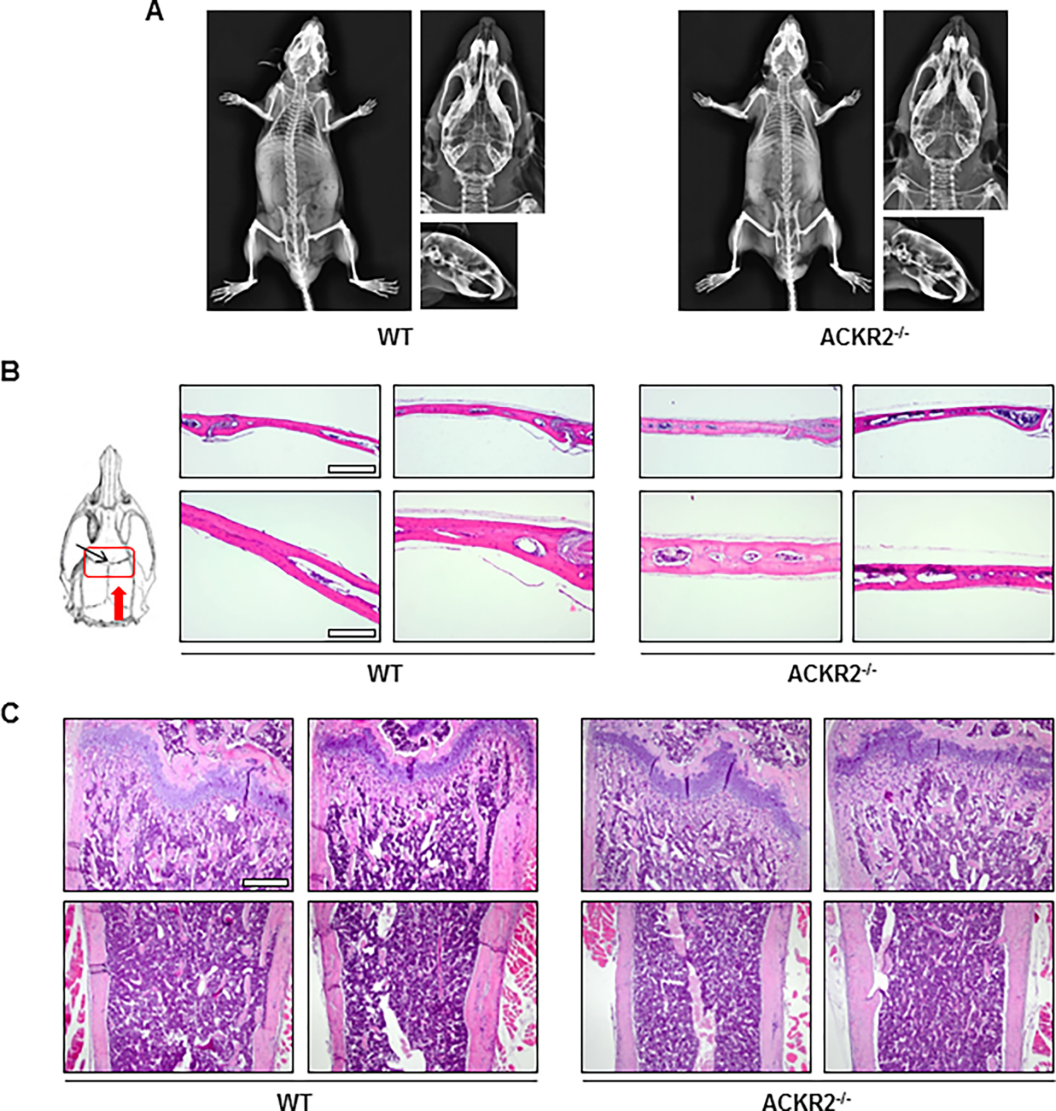
Analysis of skeletal bone and skull does not reveal overt differences in bone morphology between WT and ACKR2^-/-^ mice. (A) Representative total body (left) and cranium (right) X-ray images of WT and ACKR2^-/-^ mice. (B) Schematic representation of the skull region analysed by histology in the CHI model (red rectangle). The black arrow indicates the bregma, the red arrow the direction of cut. Representative H&E images of the skull bone histology in WT and ACKR2^-/*-*^ mice are shown on the right. Scale bar: upper panels, 500 μm; lower panels, 200 μm. (C) Representative H&E images of long bones histology in WT and ACKR2^-/^ mice. Scale bar: 500 μm.

As a possible cause of the increased frequency of death immediately after CHI we investigated the effects of ACKR2 deletion on skeletal structure, as chemokines have a well-documented role in bone physiology [41, 42]. Morphometric analysis on dorsal X-ray projection of the entire skeleton did not reveal major qualitative differences between ACKR2^-/-^ and WT mice (Fig 3A). Similarly, morphometric analysis elaborated on dorsal and lateral X-ray projections showed no significant differences on cranium diameters, aspect ratio, and roundness (Fig 3A, Table 2). Finally, H&E staining also revealed no major differences between ACKR2^-/-^ and WT mice either in the skull, analysed in the region involved in the CHI model (Fig 3B), or in long bones (Fig 3C). Overall, these results ruled out the possibility that alterations in the bone structure of ACKR2^-/-^ mice could account for their increased immediate mortality rate and altered lesion volume evolution.

**Table 2:** Skull morphometric analysis in WT and ACKR2^-/-^ mice.

Parameter	WT	ACKR2^-/-^	p value
Zygomatic width (ZW), cm	1.120 ± 0.030	1.107 ± 0.007	0.28
Braincase width (BW), cm	1.305 ± 0.034	1.307 ± 0.021	0.91
Condylobasal length (CBL), cm	2.375 ± 0.058	2.346 ± 0.087	0.51
ZW/CBL	0.472 ± 0.018	0.472 ± 0.017	0.96
Area, cm^2^	1.034 ± 0.030	1.027 ± 0.027	0.64
Perimeter, cm	5.404 ± 0.276	5.348 ± 0.194	0.67
Major axis, cm	1.847 ± 0.051	1.826 ± 0.039	0.42
Minor axis, cm	0.714 ± 0.026	0.715 ± 0.015	0.92
Circularity	0.448 ± 0.043	0.452 ± 0.031	0.81
Aspect ratio	2.593 ± 0.148	2.560 ± 0.090	0.62
Roundness	0.387 ± 0.023	0.391 ± 0.014	0.66

Morphometric analysis was based on dorsal X-ray projections on WT and ACKR2^-/-^ mice (see Fig 3A). Cranium measurements were obtained using the Image J software including: vault widths (the braincase width (BW) was measured at the skull inferior region near the paraoccipital process, the zygomatic width (ZW) was measured in the mid-vertical region at the zygomatic root of temporal bone), mid-sagittal condylobasal diameter (CBL), area, perimeter, major axis, minor axis, and circularity (1 = perfect circle to 0 = elongated shape). Cranium aspect ratio (ratio major axis/minor axis), roundness (1/aspect ratio), and the ZW/CBL ratio were calculated. For each parameter, values are reported as means ± SD.

### ACKR2 is induced acutely in the injured brain and in cultured astrocytes after inflammatory challenge

**Fig 4:**
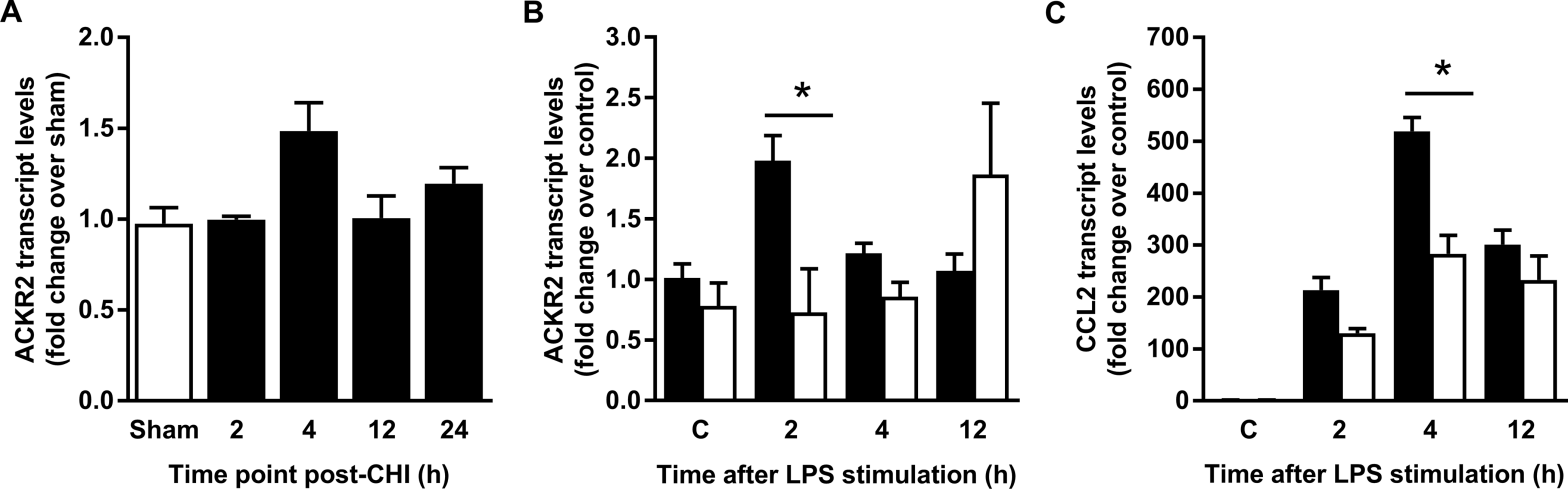
Regulation of ACKR2 and CCL2 following CHI and in *ex vivo* inflamed astrocytes. (A) ACKR2 transcript level was detected by qPCR in brain tissue of WT animals at indicated time points after CHI. Animal numbers per time point: WT sham n = 10; WT CHI 2h n = 7; WT CHI 4h n = 13; WT CHI 24h n = 6. (B) Following LPS challenge there was no change in ACKR2 expression in cultured astrocytes from WT (black columns) or CCL2^-/-^ (white columns) animals across the time points, although post-hoc analysis detected a significant difference in ACKR2 expression at 2 h (* = p < 0.05), with higher levels in WT compared to CCL2^-/-^ astrocytes. (C) CCL2 transcript level increased significantly with time in both WT (black columns) and ACKR2^-/-^ (white columns) astrocytes, with peak expression seen 4 h after LPS challenge. At all time points the expression of CCL2 was lower in ACKR2^-/-^ astrocytes as compared to WT cells. * = p < 0.05.

We next assessed changes in ACKR2 mRNA expression levels in cortical tissue at different time points after CHI (Fig 4A). As compared to sham operated control C57/BL6 mice, CHI animals showed a significant effect of ACKR2 expression over time (p = 0.04). Interestingly, ACKR2 mRNA was highest at early time points (4 h) post-CHI, though *post hoc* analyses by Tukey’s HSD did not find any difference when time points were compared with one another.

The reciprocal relationship of ACKR2 and CCL2 expression was investigated in cultured astrocytes to elucidate possible regulatory mechanisms of this ligand-receptor pair. Notably, astrocytes were chosen as they are the major producers of CCL2 allowing us to study the relationship between the chemokine receptor ACKR2 and one of the chemokines that is mostly relevant for driving macrophage/microglia recruitment to the injured brain during the neuroinflammatory processes following TBI. In addition, astrocytes have been previously reported to express ACKR2 [33, 43]. For these *in vitro* experiments, we adopted LPS stimulation as an inflammatory paradigm. As shown in Fig 4B, qPCR analysis confirmed the expression of ACKR2 transcript in WT astrocyte cultures and revealed an increase at 2 h after LPS exposure, which ANOVA demonstrated to be significant (p = 0.005). Unexpectedly, an induction of ACKR2 transcripts was not observed in CCL2^-/-^ astrocytes (Fig 4B).

Consistent with our previous data [39], LPS challenge rapidly induced the CCL2 mRNA expression in WT astrocytes. When WT and ACKR2^-/-^ astrocytes were compared, ANOVA showed a significant effect of time post-LPS (p < 0.01) with CCL2 mRNA levels peaking in both groups at 4 h, but unexpectedly the expression of CCL2 was significantly lower in ACKR2^-/-^ compared to WT cells (2-way ANOVA, p < 0.01). Additionally, 2-way ANOVA demonstrated a significant interaction between strain and time-point (p < 0.01), suggesting that the lack of ACKR2 affects the time-course of CCL2 mRNA expression in astrocytes, particularly at 4 h post-LPS stimulation (Fig 4C).

### ACKR2 deletion does not affect macrophage recruitment in experimental CHI

**Fig 5:**
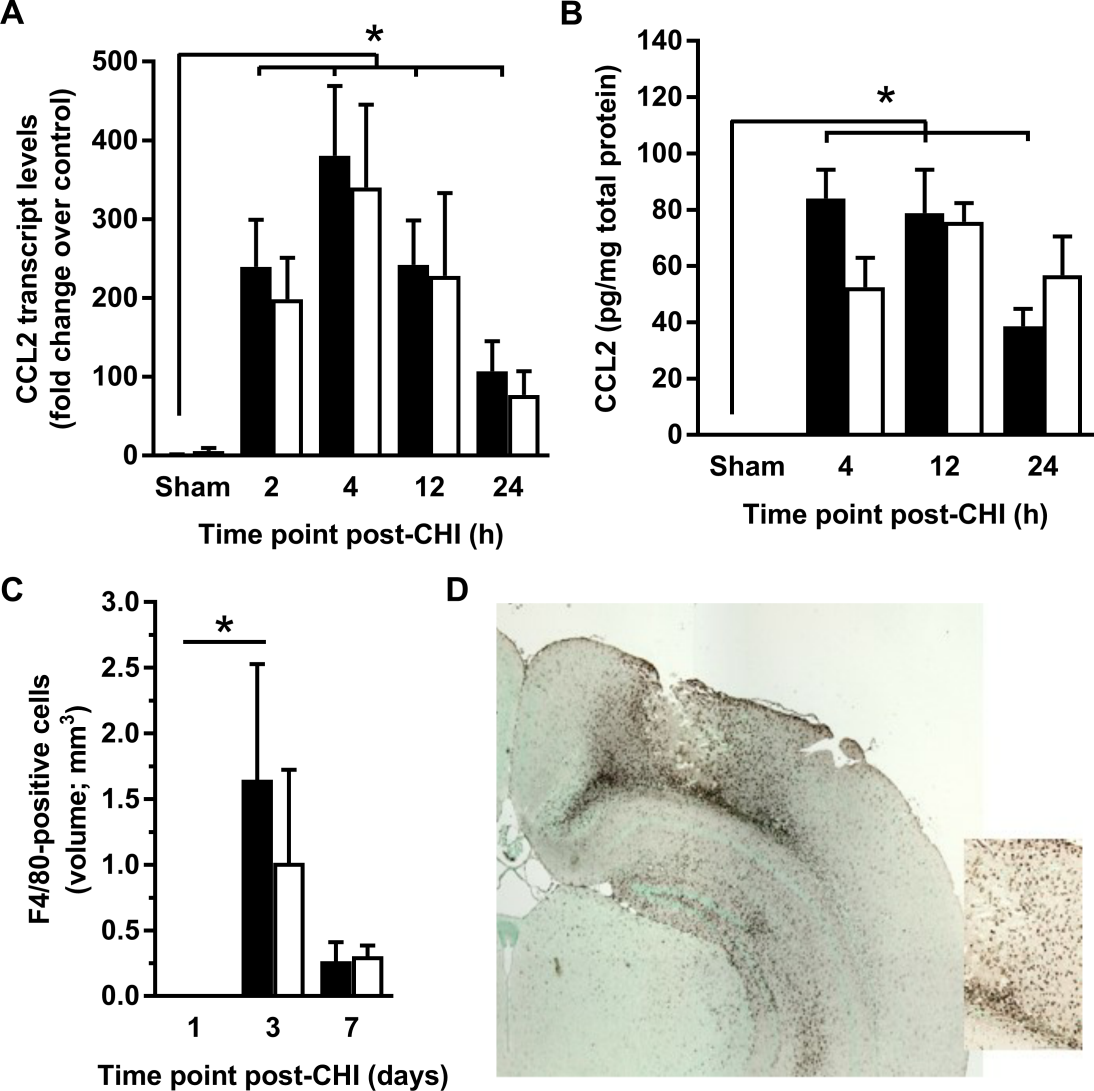
Macrophage recruitment does not differ in ACKR2^-/-^ and WT mice. (A-B) CCL2 transcript (A) and protein (B) levels were assessed in WT (black columns) and ACKR2^-/-^ mice (white columns) at indicated time points after CHI (n = 4–10), or 2 h after sham surgery (n = 4). CCL2 transcript and protein levels were significantly upregulated at all time-points assessed compared to sham levels (p < 0.01 at all time points). AVOVA detected no difference between genotypes, but multiple comparisons revealed a significant reduction in CCL2 levels between 12 and 24 h in WT (p < 0.05) but not ACKR2^-/-^ mice. (C) The volume of F4/80-positive cells was measured in the injured cortex at indicated time points. ANOVA detected no difference between genotypes at any time point analysed. (D) Accumulation of activated, F4/80-positive macrophages and microglia at 3 days post-CHI shown at low power and higher magnification (inlet). There are macrophages with a round, amoeboid morphology and activated microglia, with typical processes extending from the cell body (scale bar = 500 μm).

CCL2 mRNA expression in the pericontusional cortex following CHI showed a very similar profile in WT and ACKR2^-/-^ mice, with peak levels at 4 h after injury in both animal groups (Fig 5A). As expected, 2-way ANOVA analysis confirmed a significant effect of time (p < 0.01), but also showed that there was no effect of genotype (p = 0.69) and no significant interaction (p = 0.99).

The time-course of CCL2 protein concentration in pericontusional cortex closely matched CCL2 mRNA expression, consistent with our previous findings in this model [6] (Fig 5B). ANOVA showed a significant effect of time on CCL2 protein concentration (p < 0.01), and *post hoc* analysis by Tukey’s HSD confirmed that CCL2 protein concentration in CHI mice of either strain is significantly higher than that in sham mice at all time-points. Interestingly, although CCL2 protein expression was significantly reduced in WT mice at 24 h relative to 12 h post-CHI (p < 0.05), there was no corresponding reduction in CCL2 protein in ACKR2^-/-^ mice. Further analysis revealed no significant overall effect of genotype (p = 0.57) and no significant interaction (p = 0.10).

Concordant to our published work [6, 20], abundant recruitment of macrophages and activated microglia to the site of injury was observed, with a peak at 3 days post-injury (Fig 5C & 5D) and a significant time-point difference by ANOVA (p = 0.05). However, the volume of F4/80-positive cells showed no distinction between ACKR2^-/-^ and WT genotypes. When we examined the relationship between the volume of accumulated F4/80-positive cells and the volume of the cortical lesion based on H&E staining, a correlation was found in WT mice (3 & 7 day volumes combined, n = 8) (Pearson r = 0.7193, p = 0.0443). An even stronger correlation was detected in ACKR2^-/-^ mice (3 & 7 day volumes combined, n = 6) (Pearson r = 0.9934, p<0.0001).

### Functional recovery after CHI is not altered by ACKR2 deletion

**Fig 6:**
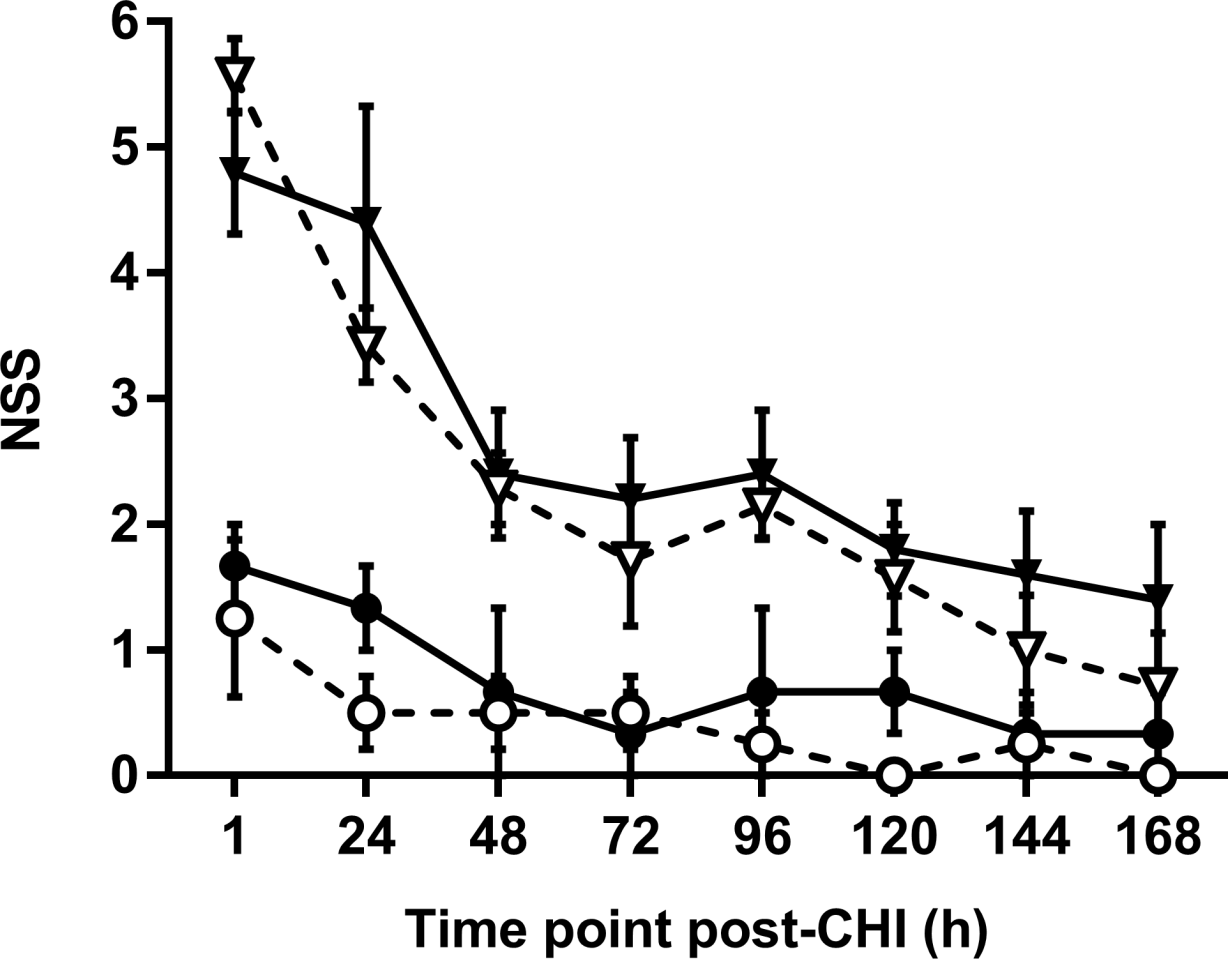
Functional recovery is similar in WT and ACKR2^-/-^ mice over 7 days post-CHI. Neurological testing was conducted for four groups of mice at indicated time points following CHI or sham surgery. WT mice (full lines) received CHI (black triangles; n = 5) or sham surgery (black dots; n = 3), ACKR2^-/-^ mice (dotted lines) were also assigned to either CHI (white triangles; n = 7) or sham surgery (white dots; n = 4). Sham animals did not show any significant sign of neurological deficit. ANOVA confirmed a significant effect of time-point on NSS in CHI animals, but did not detect difference between genotypes in functional recovery.

Based on ACKR2’s ability to sequestrate CCL2 and other inflammatory CC chemokines and the evidence from our findings of improved tissue damage and neurological function in CCL2^-/-^ mice, we hypothesised that ACKR2 deletion is detrimental to the injured brain. In order to test the hypothesis that ACKR2^-/-^ mice may have enhanced neurological deficits after injury, we assessed initial sensorimotor outcomes and ongoing recovery using the NSS over 7 days post-CHI (Fig 6). Data were analysed by 2-way repeated measures ANOVA, and as expected showed a significant effect of time (p < 0.01), a significant interaction (p < 0.01), and a significant effect of group (p < 0.01). These results indicate that: i) there is a spontaneous recovery over time; ii) sham-operated and injured mice differ in their recovery profiles; iii) injured mice of each strain have different NSS scores compared to their respective sham mice. However, there was no difference between strains when comparing NSS in the two CHI groups (p = 0.89). Individual comparisons by Tukey’s HSD only showed that injured animals performed significantly worse than their sham equivalents (p < 0.05).

## Discussion

The ability of ACKR2 in resolving inflammation has been well characterised in a number of peripheral inflammation models. Experimental evidence also suggests that ACKR2 may play a different role in different organs, though at present data are sparse and ambiguous [27, 28, 44–46]. In particular, the function of ACKR2 in inflammatory conditions of the nervous system remains obscure. Particularly in the brain, the data regarding the expression and function of ACKR2 is conflicting. The initial description of ACKR2 in 1997 showed expression in mouse brain [47], but prior to our study no detection was reported in human brain [48]. Two subsequent studies have demonstrated ACKR2 immunoreactivity in primary human astrocytes [33, 43], whereas others failed to confirm ACKR2 mRNA in cultured mouse astrocytes and did not detect uptake of fluorescently labelled CCL2 by these cells [49].

We previously demonstrated a profound upregulation in the chemokine network in both rodents and human victims suffering from severe TBI, which was associated with increased brain pathology, inflammatory cell accumulation, neuronal death and sensorimotor deficits. Based on these data, we hypothesized that following brain injury ACKR2^-/-^ mice would exhibit enhanced cerebral inflammatory responses. We thus embarked on this study to deepen our understanding on how TBI may impact on ACKR2 expression, as well as defining the potential role of this chemokine scavenger receptor in this setting. Here we demonstrate for the first time a constitutive, low ACKR2 mRNA expression in normal human brain, followed by a clear increase in ACKR2 upregulation over 10 days after TBI. Importantly, we found a significant correlation of elevated ACKR2 expression and time after injury. This data suggests a putative role for this atypical chemokine receptor in brain responses to injury. It has to be acknowledged that human TBI is a highly heterogeneous disease, a factor that may explain the large variability in the values of ACKR2 expression in post mortem brains and therefore the lack of significance between different time points. When examining the parallel changes of one of the major ligands to ACKR2, CCL2, we detected a significant and large-scale increase in CCL2 mRNA in human brain taken from victims that survived more than 6 h after TBI. There was also a significant upregulation of CCL2 mRNA in tissue collected contralateral to the site of injury, suggestive of a profound, global inflammatory response in the brain.

In order to explore further the potential function of ACKR2 in TBI, we made use of ACKR2^-/-^ mice in a well-established model of CHI. The initial unexpected observation from these experiments was a significant increase in mortality rate of ACKR2^-/-^ mice immediately following CHI. Since the timeframe of this phenomenon occurred too early to be attributed to alterations in the inflammatory response mediated by the ACKR2 deficiency, we postulated that underlying developmental abnormalities could account for this effect. Considering the involvement of CCL2 in bone metabolism [41, 42, 50], we investigated the effect of ACKR2 deletion on the bone structure of ACKR2^-/-^ skeleton and cranium; however, radiological and histological analysis found no major alterations in bone morphology of ACKR2^-/-^ mice compared to WT counterparts. Subtle changes in bone density would have required a micro-computed tomography analysis and cannot be excluded, but it appears unlikely they would have been responsible for the dramatic difference in the CHI outcome between the strains.

Comparing evolution of brain lesion in animals surviving CHI, we observed that ACKR2^-/-^ mice had significantly larger contusion volume at 1 day compared to WT mice, suggesting a potential and substantial contribution for ACKR2 in early inflammatory events after TBI. We then investigated the changes in ACKR2 transcripts relative to lesion volume progression, and found approximately a 50% increase in ACKR2 mRNA expression in WT mice versus uninjured sham animals at 4 h after injury, indicating that ACKR2 is rapidly upregulated following CHI.

Several studies have demonstrated that ACKR2 is expressed by a range of hemopoietic cells such as B-cells, T-cells, neutrophils, and macrophages [51, 52], many of which are known to infiltrate the injured brain. In the CNS, there is good evidence for ACKR2 expression in astrocytes [33, 43, 49], which represent a likely source for the increase in ACKR2 expression detected in the injured brain. As astrocytes are also the main producers of chemokines in the CNS, we investigated the expression of ACKR2 and CCL2 in cultured mouse astrocytes in response to LPS, a commonly used *in vitro* inflammatory stimulus [53, 54]. We then employed primary astrocyte cultures as an inflammatory model setting to explore regulatory mechanisms of ACKR2 expression. Interestingly, consistent to our data on the CHI model, mouse astrocytes constitutively express low levels of ACKR2. However, as early as 2 h after exposure to LPS, ACKR2 transcripts increased by 2-fold, suggesting that these cells contribute to the ACKR2 upregulation measured in brain homogenates [47]. Unfortunately, we could not establish changes of ACKR2 at protein level since the anti-human ACKR2 polyclonal antibodies commercially available does not allow for reliable immunohistochemical detection of ACKR2 in mouse tissues. Finally, it should be noted that the changes in ACKR2 trafficking properties are also relevant for its scavenger function [55], a regulatory mechanism not investigated in this study.

As the role of ACKR2 in sequestering chemokines is well established, we hypothesised that the deletion of ACKR2 would result in an amplified concentration of soluble chemokines in the cortical tissue surrounding the lesion. We measured levels of the chemokine CCL2, which is recognized and efficiently scavenged by ACKR2 and plays a key role in neuroinflammation post TBI. Indeed, CCL2 tight regulation is vital to control the inflammatory response to injury as it is expressed within hours of TBI by cells of the CNS such as astrocytes, microglia, and infiltrating macrophages [56]. Furthermore, elevated CCL2 release was shown to correlate well with macrophage accumulation [50], lesion volume progression and neurological deficits [6, 57, 58]. Consistent with previous data from us and others, we found a robust increase in CCL2 at transcript and protein levels in both WT and ACKR2^-/-^ animals following CHI. There was no significant difference between the two genotypes in terms of CCL2 mRNA or CCL2 protein, which peaked in both genotypes at 12 h post-CHI followed by a marked drop at later time point. Of note however, ACKR2^-/-^ mice showed a tendency to maintain a prolonged elevation of CCL2 at 24 h post-CHI as compared to WT mice, though this trend did not reach statistical significance.

We then examined reciprocal regulation of CCL2 and ACKR2 by measuring CCL2 transcript levels in WT and ACKR2^-/-^ astrocytes and ACKR2 transcripts in WT and CCL2^-/-^ astrocytes. As expected, a substantial upregulation of CCL2 transcripts was detected in WT astrocytes stimulated with LPS, while surprisingly ACKR2^-/-^ astrocytes displayed significantly lower levels of CCL2 transcript. Consistent with ACKR2 induction observed *in vivo* after CHI, WT astrocytes showed a significant upregulation of ACKR2 transcripts at 2 h post-LPS challenge, while in CCL2^-/-^ astrocytes ACKR2 expression was not affected. This result conflicted with our initial hypothesis and suggests that in astrocytes ACKR2 and its ligand CCL2 may set in place positive regulatory loops. Interestingly, temporal profiles of ACKR2 transcript induced *in vivo* after CHI and *in vitro* in cultured astrocytes upon LPS stimulation were similar, corroborating the potential role of astrocytes in ACKR2 expression after brain injury.

Finally, to test for any potential functional impact of ACKR2 in the pathophysiology of brain injury, we assessed the sensorimotor function in WT and ACKR2^-/-^ mice over 7 days post-CHI by using the NSS battery of tests. The NSS confirmed that both strains of mice suffered initial deficits following CHI, but no difference was demonstrated in functional recovery between ACKR2^-/-^ and WT mice. Taken together, this data indicates that ACKR2 certainly plays a role in the early inflammatory response occurring after TBI. However, this early function may be compensated in the long term as acute differences in lesion volume between ACKR2^-/-^ and WT mice vanish at late time-points, thus not affecting functional recovery.

When considering the overall impact of ACKR2 on cerebral immune responses, some aspects of its biology should be carefully considered. Firstly, ACKR2 expression, while present in the brain, is of lower magnitude compared to other barrier tissues, such as the lung, placenta, and gut [27, 28, 45]. Secondly, abundant evidence corroborated some level of redundancy in the function of chemokines in inflammatory responses [59]. A potential compensatory scavenging activity could play a role as other signalling chemokine receptors themselves have been shown to scavenge chemokines [60]. In this context, it is also possible that ACKR2 itself regulates other chemokines and chemokine receptors. Indeed, there is experimental evidence that ACKR2 suppresses the expression of other chemokine receptors, including CCR1 [61–63]. Finally, we may not exclude other unknown functions of ACKR2 on brain resident astrocytes, which require further investigations.

Previous studies have reported unexpected findings in ACKR2^-/-^ mice when tested in some disease models, including protection from experimental autoimmune encephalomyelitis [33] and reduced renal inflammation in diabetic nephropathy [64]. An emerging hypothesis is that ACKR2 regulates leukocyte interactions with lymphatic endothelial cells, which are known to express abundantly ACKR2. By clearing chemokines from the surface of lymphatic endothelial cells, ACKR2 could limit the adherence of inflammatory cells to lymphatic vessels. Indeed, impaired migration of immune cells in ACKR2^-/-^ mice has been observed [34], and recent evidence shows that lymphatic vessels are present in the brain [65]. Though in our study the amount of recruited macrophages around the cortical lesion was not different between ACKR2^-/-^ and WT mice, the potential role of this mechanism following CHI remains an attractive hypothesis to be investigated.

## Conclusion

Here we report that ACKR2 is expressed in mouse and human brain and is transiently upregulated at transcriptional level following TBI. Unexpectedly, the deletion of ACKR2 expression in gene-targeted animals had no impact on local concentrations of the inflammatory chemokine CCL2, possibly due to alternative compensatory mechanisms of the complex chemokine/chemokine receptor network, and had no relevance for functional recovery. However, at early time-points following CHI, ACKR2-knockout animals showed a significant increased mortality rate and larger lesion volumes, suggesting a distinct role for ACKR2 in the neuroinflammatory processes occurring in the injured brain compared to its reported functions in peripheral tissues.
